# Foot and ankle surgery in Australia: a descriptive analysis of the Medicare Benefits Schedule database, 1997–2006

**DOI:** 10.1186/1757-1146-1-10

**Published:** 2008-09-15

**Authors:** Hylton B Menz, Mark F Gilheany, Karl B Landorf

**Affiliations:** 1 Musculoskeletal Research Centre, Faculty of Health Sciences, La Trobe University, Bundoora, Victoria 3086, Australia; 2 Department of Podiatry, Faculty of Health Sciences, La Trobe University, Bundoora, Victoria 3086, Australia

## Abstract

**Background:**

Foot and ankle problems are highly prevalent in the general community and a substantial proportion of people seek surgical treatment to alleviate foot pain and deformity. However, the epidemiology of foot and ankle surgery has not been examined in detail. Therefore, the aim of this study was to examine patterns and costs of private sector foot surgery provision in Australia.

**Methods:**

Data pertaining to all foot and ankle surgical procedures for the calendar years 1997–2006 were extracted from the Australian Medicare Benefits Schedule (MBS) database and were cross-tabulated by sex and age. Descriptive analyses were undertaken to assess sex and age differences in the number and type of procedures performed and to assess for temporal trends over the ten year assessment period. The total cost to Medicare of subsiding surgeons' fees in 2006 was also determined.

**Results:**

During the 1997–2006 period, 996,477 surgical procedures were performed on the foot and ankle by private surgeons in Australia. Approximately equal numbers of procedures were performed on males (52%) and females (48%). However, males were more likely to undergo toenail, ankle, clubfoot, tarsal coalition and congenital vertical talus surgery, whereas females were more likely to undergo lesser toe, first metatarsophalangeal joint (MPJ), neuroma, heel, rearfoot and lesser MPJ surgery. The total number of procedures was stable over the assessment period, however there was a relative increase in the number of procedures performed on people aged over 55 years. The total contribution of Medicare to subsiding surgeons' fees for procedures performed in 2006 was $14 M.

**Conclusion:**

Foot and ankle surgery accounts for a considerable degree of healthcare expenditure in Australia, and the number of procedures in those aged over 55 years is increasing. Given the ageing demographics of the Australian population, the future public health and economic impact of foot morbidity is likely to be substantial. Strategies need to be implemented to ensure that the surgical labour force is adequate to address this increasing demand.

## Background

Foot problems are reported by at least one in five people in the general population [1,2], and are associated with self-reported disability [3] and reduced health-related quality of life [2,4,5]. Although many common foot problems can be effectively managed by conservative interventions such as lesion debridement, physiotherapeutic modalities, orthotic therapy and footwear modifications, major structural or long-standing conditions often require surgical intervention. In Australia, provision of foot surgery is primarily the domain of specialist orthopaedic surgeons. However, general surgeons and general practitioners may also perform foot and ankle surgical procedures, and a small number of surgically-trained podiatrists perform forefoot surgery in the private sector [6,7].

Foot and ankle surgery in Australia is heavily subsidised by Medicare, a universal healthcare system financed through income tax and an income-related Medicare levy. Governed by the Australian Health Care Agreements between the Commonwealth and the states, Medicare covers the full cost of procedures performed by public surgeons in public hospitals, and 75% of the scheduled fee for procedures performed by private surgeons. Additional private hospital costs (such as theatre fees) are not covered by Medicare, and are generally met by private health insurance [8]. Podiatric surgeons do not attract a Medicare subsidy, however several private health insurance funds provide rebates for their services [6].

Surgical activity in Australia is continuously tracked by the Medicare Benefits Schedule (MBS) database [9], which records all services performed by registered providers that qualify for a Medicare benefit, with the exception of: (i) services provided by hospital doctors to public patients in public hospitals; (ii) services that qualify for a benefit under the Department of Veterans' Affairs, Work Cover or the Transport Accident Commission, and; (iii) services provided by podiatric surgeons. Although not completely comprehensive, the MBS database is nevertheless a useful resource for exploring the epidemiology and healthcare costs of surgical procedures [10].

The aim of this study was to investigate patterns of foot and ankle surgery provision in Australia, with particular reference to: (i) the influence of age and sex on the type of surgery performed; (ii) temporal trends in surgical provision over a ten-year period (1997–2006), and; (iii) the total cost to Medicare of subsiding foot and ankle surgeons' fees in 2006.

## Methods

### Data extraction from the Medicare Benefits Schedule database

Data pertaining to foot and ankle surgical procedures for the calendar years 1997–2006 were extracted from the publicly accessible Medicare Benefits Schedule (MBS) database [9]. A summary of the item numbers and procedures obtained is provided in Additional File 1. Each item number dataset (consisting of the number of procedures performed by sex, age-group and calendar year) was extracted individually and exported into Microsoft Excel (Microsoft Corp, Redmond USA) for analysis. MBS item numbers for procedures that could not be isolated to the foot and ankle (such as excision of soft tissue tumours, treatment of burns, "generic" surgical items and multilevel orthopaedic surgery) were excluded.

Costs per item number (in Australian dollars) were obtained for the 2006 calendar year only, as the cost data did not encompass the entire assessment period (1997–2006).

To simplify the interpretation of the results, item numbers relating to a similar region of the foot or a specific foot condition were combined into one of the following 12 categories:

(i) toenail: wedge resection, partial resection and total removal of toenails (item numbers 44136, 47904, 47906, 47912, 47915, 47916, 47918);

(ii) foot and ankle trauma: including treatment of dislocations and fractures of the ankle, tarsals, metatarsals and phalanges (item numbers 47063, 47066, 47069, 47072, 47594, 47597, 47600, 47603, 47606, 47609, 47612, 47615, 47618, 47621, 47624, 47627, 47630, 47633, 47636, 47639, 47642, 47645, 47648, 47651, 47654, 47657, 47663, 47666, 47672, 47678);

(iii) lesser toes: including primary and secondary repair of flexor and extensor tendons, tenotomy, correction of clawtoes, hammertoes and hyperextension deformity (item numbers 49800, 49803, 49806, 49809, 49812, 49848, 49851 and 50345);

(iv) ankle: including diagnostic arthroscopy, arthroscopic surgery, ligamentous stabilisation, arthrodesis, total joint replacement and Achilles tendon procedures (item numbers 49700, 49703, 49706, 49709, 49712, 49715, 49718, 49721, 49724, 49727 and 50312);

(v) first metatarsophalangeal joint (1^st ^MPJ): including excisional arthroplasty, osteotomy, adductor hallucis tendon transfer, prosthetic arthroplasty and arthrodesis for either hallux valgus or hallux rigidus (item numbers 49821, 49824, 49827, 49830, 49833, 49836, 49837, 49838, 49839, 49842, 49845, 49857, 49860 and 49863);

(vi) neuroma: neurectomy for plantar digital neuritis (item number 49866);

(vii) amputations: digital, transmetatarsal, Syme (item numbers 44338, 44342, 44346, 44350, 44354, 44358, 44359, 44361, 44364);

(viii) clubfoot: including posterior release, medial release or combined postero-medial release (item numbers 50315, 50318, 50321, 50324, 50327, 50339 and 50342);

(ix) heel: including excision of calcaneal spur and plantar fasciotomy (item numbers 49818 and 49854);

(x) rearfoot: including triple arthrodesis and subtalar joint arthrodesis (item numbers 49815 and 50118);

(xi) lesser metatarsophalangeal joints: synovectomy of metatarsophalangeal joints (item numbers 49860 and 49863);

(xii) tarsal coalition and congenital vertical talus (item numbers 50333 and 50336);

To calculate the total number of procedures performed between 1997 and 2006, all item numbers pertaining to bilateral procedures (49824, 49830, 49836, 49838, 49842 and 50327) were considered to represent two individual procedures. However, when calculating total costs for the year 2006, the cost for each item (unilateral or bilateral) was used.

To evaluate trends in the total number of procedures per year between 1997 and 2006, both absolute and adjusted figures were calculated, as approximately two million additional people were enrolled in Medicare over the assessment period. To determine the number of procedures relative to the number of people enrolled, the number of eligible people for the last quarter of each year was extracted from the database, and the number of procedures was expressed per 100,000. However, because the database does not report sex or age of those enrolled, it was not possible to determine whether trends over time differed according to these characteristics.

### Statistical analysis

Data were analysed using simple descriptive statistics (total number of procedures cross-tabulated by sex, age-group and calendar year), as the publicly accessible version of the MBS database we used does not allow for the extraction of individual-level data. For each category of procedures, the proportion of item numbers documented for males and females was determined and expressed as a ratio.

## Results

### All procedures

**Figure 1:**
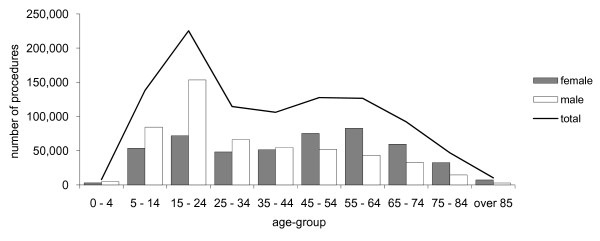
**Total number of surgical procedures performed between 1997 and 2006 according to age and sex**.

**Figure 2:**
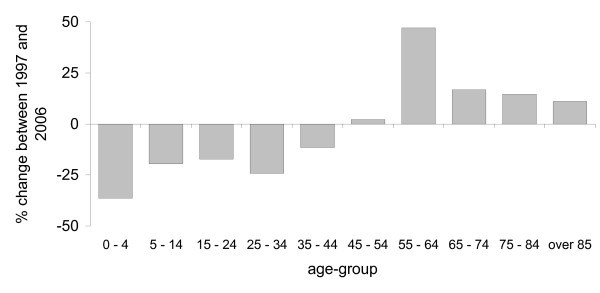
Percentage change in the total number of procedures performed between 1997 and 2006 according to age.

Using our audit methodology, between 1997 and 2006, a total of 996,477 surgical procedures attracting a Medicare rebate were performed on the foot and ankle by private surgeons (excluding podiatric surgeons) in Australia. The most frequently performed surgical category was toenail surgery (64%), followed by trauma (16%), lesser toe (6%) and ankle (6%) surgery. Approximately equal numbers of procedures were performed on males (52%) and females (48%). The distribution of procedures according to age demonstrated two peaks: one for the 15 to 24 year age-group and one for the 55 to 64 year age-group (see Figure 1). Between 1997 and 2006, the total number of procedures performed remained reasonably stable (ranging from 94,217 to 104,538 procedures per year, or 81 to 124 procedures per year per 100,000 people enrolled). However, there was a relative decrease in the number of procedures in those aged 0 to 44 years, and a relative increase in those aged 45 years and over (see Figure 2).

### Toenail procedures

**Figure 3:**
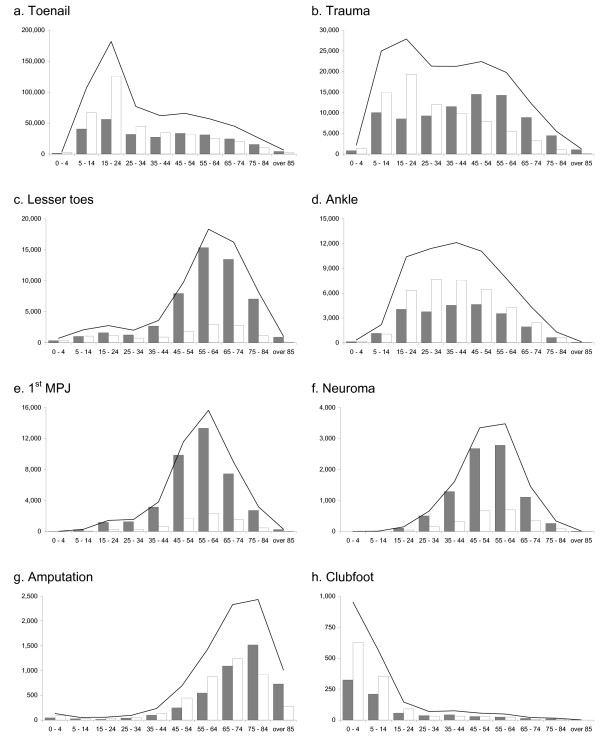
Total number of procedures by surgical category between 1997 and 2006 according to age and sex (females – grey bars, males – white bars, total – line).

A total of 630,744 surgical procedures were performed on toenails, with a male to female ratio of 1.39. The highest proportion of procedures was performed on the 15 to 24 year age group (see Figure 3a).

### Trauma procedures

A total of 158,604 surgical procedures were performed for foot and ankle trauma, with a male to female ratio of 1.09. The highest proportion of procedures was performed on the 15 to 24 year age group (see Figure 3b).

### Lesser toe procedures

A total of 64,764 surgical procedures were performed on the lesser toes, with a female to male ratio of 3.88. The highest proportion of procedures was performed on the 55 to 64 year age group (see Figure 3c).

### Ankle procedures

A total of 61,113 surgical procedures were performed on the ankle, with a male to female ratio of 1.51. The highest proportion of procedures was performed on the 35 to 44 year age group (see Figure 3d).

### 1^st ^MPJ procedures

A total of 46,727 surgical procedures were performed on the 1^st ^MPJ, with a female to male ratio of 5.35. The highest proportion of procedures was performed on the 55 to 64 year age group (see Figure 3e).

### Neuroma procedures

A total of 11,037 surgical procedures were performed on intermetatarsal neuromas, with a female to male ratio of 3.78. The highest proportion of procedures was performed on the 55 to 64 year age group (see Figure 3f).

### Amputation procedures

A total of 8,463 surgical procedures were performed for foot amputation, with a male to female ratio of 1.13. The highest proportion of procedures was performed on the 75 to 84 year age group (see Figure 3g).

### Clubfoot procedures

A total of 1,950 surgical procedures were performed for clubfoot, with a male to female ratio of 1.61. The highest proportion of procedures was performed on the 0 to 4 year age group (see Figure 3h).

### Heel procedures

**Figure 4:**
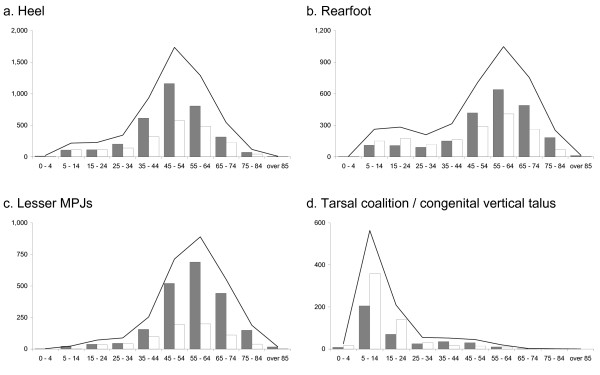
Total number of procedures by surgical category between 1997 and 2006 according to age and sex (females – grey bars, males – white bars, total – line).

A total of 5,446 surgical procedures were performed on the heel, with a female to male ratio of 1.67. The highest proportion of procedures was performed on the 45 to 54 year age group (see Figure 4a).

### Rearfoot procedures

A total of 3,855 surgical procedures were performed on the rearfoot, with a female to male ratio of 1.33. The highest proportion of procedures was performed on the 55 to 64 year age group (see Figure 4b).

### Lesser MPJ procedures

A total of 2,800 surgical procedures were performed on the lesser MPJs, with a female to male ratio of 2.87. The highest proportion of procedures was performed on the 55 to 64 year age group (see Figure 4c).

### Tarsal coalition and congenital vertical talus procedures

A total of 941 and 33 surgical procedures were performed for tarsal coalition and congenital vertical talus, respectively. Surgery for tarsal coalition and congenital vertical talus was more commonly performed on males (male to female ratios of 1.52 and 1.75, respectively). The highest proportion of procedures for both conditions were performed on people aged between 5 and 14 years of age (see Figure 4d).

### Changes over time by procedure

**Figure 5:**
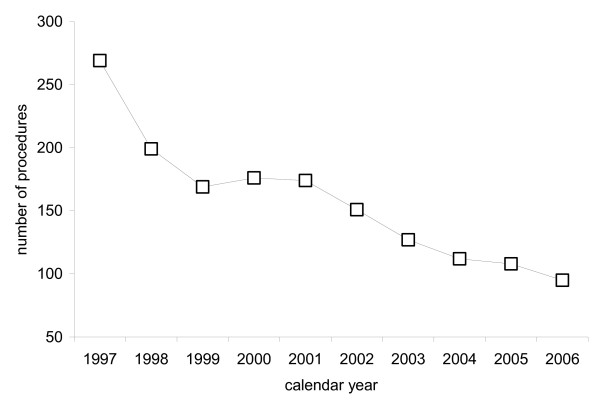
Total number of surgical procedures undertaken for the excision of calcaneal spurs (MBS item number 49818) performed per year between 1997 and 2006.

Examination of the number of procedures performed over time indicated a steady increase in the number of lesser toe, ankle, 1^st ^MPJ, botulinum toxin, rearfoot and lesser MPJ procedures, no change in the number of toenail, trauma, neuroma, amputation, clubfoot and tarsal coalition/congenital vertical talus procedures, and a steady decrease in the number of heel procedures (data not shown). Of particular note, there was a marked reduction in the number of procedures undertaken for the excision of calcaneal spurs (item number 49818). See Figure 5.

### Costs

**Figure 6:**
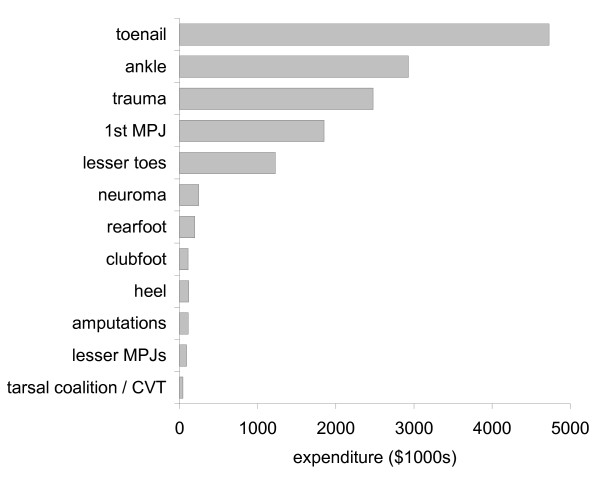
Expenditure according to procedure category in 2006. NB: 1^st ^MPJ – first metatarsophalangeal joint; CVT – congenital vertical talus.

In 2006, 96,217 foot and ankle surgery items were documented, resulting in a total Medicare contribution to surgeons' fees of $14,128,342. The highest expenditure by according to procedure type was for toenail surgery ($4.73 M, or 33% of total expenditure). A summary of total expenditure for each procedure category is shown in Figure 6.

## Discussion

The aim of this study was to explore the epidemiology and costs of foot and ankle surgery in Australia using data routinely tracked by the Medicare Benefits Schedule (MBS) database, in order to inform future planning of foot surgery provision. Before discussing the findings in detail, it is worth considering several limitations inherent in the database. First, the MBS database does not cover services provided by hospital doctors to public patients in public hospitals, services that qualify for a benefit under the Department of Veterans' Affairs, Work Cover or the Transport Accident Commission, or services provided by podiatric surgeons. As such, the data presented here cannot be considered comprehensive. Second, the publicly accessible version of the database does not delineate between types of surgical providers (e.g.: general surgeons or orthopaedic surgeons), so no analyses could be performed to compare patterns of surgical provision between these groups. Third, although foot and ankle surgery often involves multiple item numbers per procedure (e.g.: combined hallux valgus and hammertoe surgery), MBS data is recorded according to individual item codes. Therefore, the database cannot be used to ascertain the number of *patients *undergoing foot surgery. Fourth, by excluding items which are commonly documented for foot surgery but do not specifically pertain to the foot, the incidence of foot surgery reported here is an underestimate. Finally, analysis of the database was restricted to descriptive statistics, as individual-level data cannot be extracted.

Despite these limitations, our analysis of the MBS database yielded several interesting findings. Overall, nearly one million surgical procedures were performed on the foot and ankle between 1997 and 2006, and the total cost of subsidising surgeons' fees in the most recent assessment year was $14 M. By far the most commonly performed surgery type was toenail surgery, accounting for 64% of all procedures and 33% of total expenditure in 2006. Somewhat surprisingly, the total number of procedures per calendar year was reasonably stable over time, although temporal trends varied according to age and procedure type. While there appears to have been a reduction in the number of procedures performed on younger people, there has been a significant increase in the number of procedures performed on older people, particularly those aged 55 to 64 years. Consistent with this observation, the total number of procedures for conditions commonly affecting younger people (such as toenail and clubfoot surgery) decreased between 1997 and 2006, whereas procedures more commonly performed on older people (such as lesser toe and 1^st ^MPJ surgery) demonstrated significant increases over this period.

Notably, between 1997 and 2006 there was a marked reduction in the number of procedures undertaken for the excision of calcaneal spurs. In 1997, 269 such procedures were performed, and the number steadily declined to 95 in 2006. It is possible that this change reflects either an increase in the number of people seeking conservative treatment for heel pain, or a shift in surgical practice in response to: (i) research questioning the role of calcaneal spurs in the pathophysiology of heel pain [11]; or (ii) the recent availability of extracorporeal shock wave therapy [12], despite the limited evidence for its effectiveness [13].

Sex differences in the types of foot and ankle surgery undertaken were generally consistent with available literature pertaining to the prevalence of various foot disorders. Females were far more likely to undergo lesser toe, 1^st ^MPJ, neuroma and lesser MPJ surgery, which reflects the known female predisposition to these conditions [1,14,15]. In adult females, this may be explained (at least in part) by the detrimental effects of women's fashion footwear, which often has an elevated heel and narrow toebox [16]. Indeed, Coughlin and Thompson [17] have estimated that in the US in 1991, 209,000 bunionectomies, 210,000 hammer toe corrections, 119,000 bunionette repairs and 66,500 neuroma resections were performed (at a cost of approximately US$3 billion) for shoe-related foot problems. However, not all procedures were more common in females, with males being more likely to undergo toenail, ankle, clubfoot, tarsal coalition and congenital vertical talus surgery. While the epidemiological literature pertaining to these foot conditions is sparse, there is some evidence that males are more likely to develop thickened nails [18], onychomycosis [19], clubfoot [20-22] and tarsal coalition [23].

Age differences in the types of foot and ankle surgery undertaken were also consistent with the known incidence and prevalence of specific foot conditions. As would be expected, surgical treatment for congenital conditions such as clubfoot, vertical talus, tarsal coalition and equino-valgus associated with cerebral palsy were almost exclusively represented in those aged less than 10 years, whereas amputations and treatment for chronic arthritic disorders of the forefoot and rearfoot were over-represented in those aged 55 years and over. Interestingly, the age distribution of procedures for foot and ankle trauma exhibited a bimodal distribution, with one peak for the 15 to 24 age-group (with an over-representation of males), and a second peak for people aged over 55 years (with an over-representation of females). Although the underlying mechanism for the trauma requiring surgery cannot be ascertained from the database, it is likely that the first peak primarily represents sporting injuries and occupational foot and ankle trauma in young men [24,25], while the second peak may be related to osteoporotic fractures associated with accidental falls in older women [24,26].

To the authors' knowledge, there are only two similar analyses that have been reported in the literature. The first was a clinical audit of 785 cases of foot surgery conducted by ten fellows of the Australasian College of Podiatric Surgeons between July 1995 and June 1996 [7]. This study revealed that the most commonly performed procedures were for lesser toe deformities (46%), followed by hallux valgus (21%), intermetatarsal neuroma (8%) and hallux limitus/rigidus (7%). Most patients were female (80%), and the highest proportion of patients were aged 51 to 61 years. More recently, a population-based study of 6,956 inpatient cases in Sweden [27] indicated that the most common surgical procedures undertaken were for the treatment of hallux valgus (60%), followed by hammer toes (24%) and hallux limitus/rigidus (15%). Consistent with the present study findings, most of these procedures were performed on women aged 50 to 70 years of age.

The findings reported in this study have important implications for surgical labour force planning. Although the total number of procedures between 1997 and 2006 remained reasonably stable, there was a marked increase in the number of procedures performed on those aged 55 to 64 years. Population projections by the Australian Bureau of Statistics indicate that, due to declining birth and death rates and the transition of the so-called "Baby Boomer" generation into retirement age, Australia will undergo a significant ageing of the population over the next 50 years. In fact, by 2051, it is estimated that the number of people aged over 65 years will double, and the number of people aged over 85 years will quadruple as a relative proportion of the total population [28].

These demographic changes will undoubtedly result in a significant increase in the number of older people requiring surgical treatment for foot disorders, which will necessitate a corresponding increase in the surgical workforce to meet this need. In addition to training more orthopaedic surgeons, meeting this demand may require alternative strategies such as task substitution, whereby foot surgery is undertaken by other healthcare professionals such as podiatrists. Although the role of podiatric surgeons is well established in the US and UK, only recently has orthopaedic task substitution been suggested in Australia [29]. Given that there is some evidence from the US that podiatric surgery is less expensive than orthopaedic foot surgery (due to fewer hospital admissions [30]) and that podiatric surgery in the UK reduces the need for ongoing podiatry treatment [31], there may also be cost savings associated with broader integration of podiatrists into the Australian surgical workforce. This may be of particular relevance to toenail surgery, as simple nail avulsion with phenolisation (commonly performed under local anaesthesia by podiatrists) has been shown to be more effective at preventing recurrence than surgical excision techniques favoured by orthopaedic and general surgeons [32]. However, such role flexibility may also be extended to forefoot surgery, as evidence from the UK indicates high levels of satisfaction (in both patients [33] and referring general practitioners [34]) with podiatric surgeons undertaking these procedures.

## Conclusion

Foot and ankle surgery accounts for a considerable degree of healthcare expenditure in Australia. Given the ageing demographics of the Australian population, the future public health and economic impact of foot morbidity is likely to be substantial. Strategies need to be implemented to ensure that the surgical labour force is adequate to address this increasing demand.

## Competing interests

HBM and KBL are Editor-in-Chief and Deputy Editor-in-Chief, respectively, of the *Journal of Foot and Ankle Research*. It is journal policy that editors are removed from the peer review and editorial decision making processes for papers they have co-authored.

## Authors' contributions

HBM and MFG conceived the study. HBM extracted, analysed and interpreted the data, and drafted the manuscript. MFG and KBL assisted with data interpretation. All authors read and approved the final version of the manuscript.

## Supplementary Material

Additional file 1 Click here for file
