# Supplementary material for: Foot and ankle surgery in Australia: a descriptive analysis of the Medicare Benefits Schedule database, 1997–2006
Source: J Foot Ankle Res. 2008 Sep 15;1:10. doi: 10.1186/1757-1146-1-10 (PMC2553783; doi:10.1186/1757-1146-1-10)
Supplement: Additional file 1 [file 1757-1146-1-10-S1.doc]

| Item | Procedure description |
| --- | --- |
| 44136 | Ingrown toenail, operation for, under general anaesthesia |
| 44342 | 1 digit of foot, amputation of |
| 44342 | 2 digits of 1 foot, amputation of |
| 44346 | 3 digits of 1 foot, amputation of |
| 44350 | 4 digits of 1 foot, amputation of |
| 44354 | 5 digits of 1 foot, amputation of |
| 44358 | Toe, including metatarsal or part of metatarsal each toe, amputation of |
| 44359 | One or more toes of one foot, amputation of, including if performed, excision of 1 or more metatarsal bones of the foot, performed for diabetic or other microvascular disease |
| 44361 | Foot at ankle (Syme, Pirigoff types), amputation of |
| 44364 | Foot, midtarsal or transmetatarsal, amputation of |
| 47063 | Ankle or tarsus, treatment of dislocation of, by closed reduction |
| 47066 | Ankle or tarsus, treatment of dislocation of, by open reduction |
| 47069 | Toe, treatment of dislocation of, by closed reduction |
| 47072 | Toe, treatment of dislocation of, by open reduction |
| 47594 | Ankle joint, treatment of fracture of, not being a service to which item 47597 applies |
| 47597 | Ankle joint, treatment of fracture of, by closed reduction |
| 47600 | Ankle joint, treatment of fracture of, by internal fixation of 1 of malleolus, fibula or diastasis |
| 47603 | Ankle joint, treatment of fracture of, by internal fixation of more than 1 of malleolus, fibula or diastasis |
| 47606 | Calcaneum or talus, treatment of fracture of, not being a service to which item 47609, 47612, 47615 or 47618 applies, with or without dislocation |
| 47609 | Calcaneum or talus, treatment of fracture of, by closed reduction, with or without dislocation |
| 47612 | Calcaneum or talus, treatment of intra-articular fracture of, by closed reduction, with or without dislocation |
| 47615 | Calcaneum or talus, treatment of fracture of, by open reduction, with or without dislocation |
| 47618 | Calcaneum or talus, treatment of intra-articular fracture of, by open reduction, with or without dislocation |
| 47621 | Tarso-metatarsal, treatment of intra-articular fracture of, by closed reduction, with or without dislocation |
| 47624 | Tarso-metatarsal, treatment of fracture of, by open reduction, with or without dislocation |
| 47627 | Tarsus (excluding calcaneum or talus), treatment of fracture of |
| 47630 | Tarsus, (excluding calcaneum or talus), treatment of fracture of, by open reduction, with or without dislocation |
| 47633 | Metatarsal, 1 of, treatment of fracture of |
| 47636 | Metatarsal, 1 of, treatment of fracture of, by closed reduction |
| 47639 | Metatarsal, 1 of, treatment of fracture of, by open reduction |
| 47642 | Metatarsals, 2 of, treatment of fracture of |
| 47645 | Metatarsals, 2 of, treatment of fracture of, by closed reduction |
| 47648 | Metatarsals, 2 of, treatment of fracture of, by open reduction |
| 47651 | Metatarsals, 3 or more of, treatment of fracture of |
| 47654 | Metatarsals, 3 or more of, treatment of fracture of, by closed reduction |
| 47657 | Metatarsals, 3 or more of, treatment of fracture of, by open reduction |
| 47663 | Phalanx of great toe, treatment of fracture of, by closed reduction |
| 47666 | Phalanx of great toe, treatment of fracture of, by open reduction |
| 47672 | Phalanx of toe, (other than great toe), 1 of, treatment of fracture of, by open reduction |
| 47678 | Phalanx of toe, (other than great toe), more than 1 of, treatment of fracture of, by open reduction |
| 47904 | Digital nail of toe, removal of, not being a service to which item 47096 applies |
| 47906 | Digital nail of toe, removal of, in the operating theatre of hospital |
| 47912 | Pulp space infection, paraonychia of foot, incision for, not being a service to which another item in this group applies |
| 47915 | Ingrowing nail of toe, wedge resection for, including removal of segement of nail, ungula fold and portion of the nail bed |
| 47916 | Ingrowing nail of toe, partial resection of nail, including phenolisation but not including excision of nail bed |
| 47918 | Ingrowing toenail, radical excision of nail bed |
| 49700 | Ankle, diagnostic arthroscopy of, including biopsy |
| 49703 | Ankle, arthroscopic surgery of |
| 49706 | Ankle, arthrotomy of, involving 1 or more of: lavage, removal of loose body or division of contracture |
| 49709 | Ankle, ligamentous stabilisation of |
| 49712 | Ankle, arthrodesis of |
| 49715 | Ankle, total joint replacement of |
| 49718 | Ankle, Achilles' tendon or other major tendon, repair of |
| 49721 | Ankle, Achilles' tendon rupture managed by non-operative treatment |
| 49724 | Ankle, Achilles' tendon, secondary repair or reconstruction of |
| 49727 | Ankle, Achilles' tendon, operation for lengthening |
| 49800 | Foot, flexor or extensor tendon, primary repair of |
| 49803 | Foot, flexor or extensor tendon, secondary repair of |
| 49806 | Foot, subcutaneous tenotomy of, 1 or more tendons |
| 49809 | Foot, open tenotomy of, with or without tenoplasty |
| 49812 | Foot, tendon or ligament transplantation of, not being a service to which another item in this group applies |
| 49815 | Foot, triple arthrodesis of |
| 49818 | Foot, excision of calcaneal spur |
| 49821 | Foot, correction of hallux valgus or hallux rigidus by excision arthroplasty (Keller's or similar procedure) – unilateral |
| 49824 | Foot, correction of hallux valgus or hallux rigidus by excision arthroplasty (Keller's or similar procedure) – bilateral |
| 49827 | Foot, correction of hallux valgus by transfer of adductor hallucis tendon – unilateral |
| 49830 | Foot, correction of hallux valgus by transfer of adductor hallucis tendon – bilateral |
| 49833 | Foot, correction of hallux valgus by osteotomy of first metatarsal including internal fixation where performed – unilateral |
| 49836 | Foot, correction of hallux valgus by osteotomy of first metatarsal including internal fixation where performed – bilateral |
| 49837 | Foot, correction of hallux valgus by osteotomy of first metatarsal and transfer of adductor hallucis tendon, including internal fixation where performed – unilateral |
| 49838 | Foot, correction of hallux valgus by osteotomy of first metatarsal and transfer of adductor hallucis tendon, including internal fixation where performed – bilateral |
| 49839 | Foot, correction of hallux rigidus or hallux valgus by prosthetic arthroplasty – unilateral |
| 49842 | Foot, correction of hallux rigidus or hallux valgus by prosthetic arthroplasty – bilateral |
| 49845 | Foot, arthrodesis of, first metatarso-phalangeal joint |
| 49848 | Foot, correction of claw or hammer toe |
| 49851 | Foot, correction of claw or hammer toe with internal fixation |
| 49854 | Foot, radical plantar fasciotomy or fasciectomy of |
| 49857 | Foot, metatarso-phalangeal joint replacement |
| 49860 | Foot, synovectomy of metatarso-phalangeal joint, single joint |
| 49863 | Foot, synovectomy of metatarso-phalangeal joint, 2 or more joints |
| 49866 | Foot, neurectomy for plantar or digital neuritis (Morton's or Bett's syndrome) |
| 50118 | Subtalar joint, arthrodesis of |
| 50312 | Ankle, synovectomy of |
| 50315 | Talipes equinovarus, posterior release of |
| 50318 | Talipes equinovarus, medial release of |
| 50321 | Talipes equinovarus, combined postero-medial release of |
| 50324 | Talipes equinovarus, combined postero-medial release of, revision procedure |
| 50327 | Talipes equinovarus, bilateral procedures |
| 50333 | Tarsal coalition, excision of, with interposition of muscle, fat graft or similar graft |
| 50336 | Talus, vertical, congenital, combined anterior and posterior reconstruction |
| 50339 | Foot and ankle, tibialis anterior tendon (split or whole) transfer to lateral column |
| 50342 | Foot and ankle, tibialis or tibialis posterior tendon transfer, through the interosseous membrane to anterior or posterior aspect of foot |
| 50345 | Hyperextension deformity of toe release incorporating V-Y plasty of skin, lengthening of extensor tendons and release of capsule contracture |
